# 7-Chloro-5-cyclo­propyl-9-methyl-5*H*-4,5,6,10-tetra­aza­dibenzo[*a*,*d*]cyclo­hepten-11(10*H*)-one

**DOI:** 10.1107/S1600536811018022

**Published:** 2011-05-20

**Authors:** S. Naveen, N. R. Thimmegowda, H. R. Manjunath, M. A. Sridhar, J. Shashidhara Prasad, K. S. Rangappa

**Affiliations:** a Department of Physics, Sri Bhagawan Mahaveer Jain College of Engineering, Jain University, Bangalore 562 112, India; b Department of Studies in Chemistry, Manasagangotri, University of Mysore, Mysore 570 006, India; c Department of Studies in Physics, Manasagangotri, University of Mysore, Mysore 570 006, India; d Sri Sathya Sai Institute of Higher Learning, Prasanthi Nilayam, 515 134, India

## Abstract

In the title compound, C_15_H_13_ClN_4_O, which is a chloro derivative of the drug Nevirapine, the diazepine ring is in a twisted boat conformation. The pyridine rings fused to the diazepine fragment form a dihedral angle of 58.44 (10)° and the mol­ecule adopts a butterfly shape. The mol­ecules are joined *via* N—H⋯N hydrogen bonding into polymeric chains down the *b* axis. All weaker C—H⋯O inter­actions involve the carbonyl O atom as acceptor.

## Related literature

For background to the chemistry of azepines, see: Le Count (1996 ▶). The title compound is a chloro derivative of the anti-HIV drug nevirapine (systematic name 11-cyclo­propyl-4-methyl-5,11-dihydro-6*H*-dipyrido[3,2-*b*:2′,3′-*e*][1,4]diazepin-6-one) and was synthesised as a basic scaffold, see: Matsumoto *et al.* (1984 ▶). We have also synthesized its derivatives and tested for secretory phospho­lipase A_2_ with anti-inflammatory activity, see: Thimmegowda *et al.* (2007 ▶). For a related structure, see: Thimmegowda *et al.* (2008 ▶). For ring puckering parameters, see: Cremer & Pople (1975 ▶).
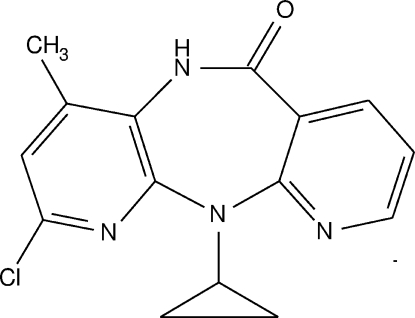


## Experimental

### 

#### Crystal data

C_15_H_13_ClN_4_O
*M* *_r_* = 300.74
Orthorhombic, 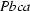

*a* = 12.7750 (6) Å
*b* = 13.5870 (7) Å
*c* = 16.4920 (9) Å
*V* = 2862.6 (3) Å^3^
*Z* = 8
Mo *K*α radiation
μ = 0.27 mm^−1^
*T* = 293 K
0.27 × 0.25 × 0.25 mm

#### Data collection

MacScience DIPLabo 32001 diffractometer
4721 measured reflections
2525 independent reflections
2155 reflections with *I* > 2σ(*I*)
*R*_int_ = 0.014

#### Refinement

*R*[*F* ^2^ > 2σ(*F* ^2^)] = 0.040
*wR*(*F* ^2^) = 0.114
*S* = 1.03
2525 reflections
192 parameters
H-atom parameters constrained
Δρ_max_ = 0.34 e Å^−3^
Δρ_min_ = −0.39 e Å^−3^

### 

Data collection: *XPRESS* (MacScience, 2002 ▶); cell refinement: *SCALEPACK* (Otwinowski & Minor, 1997 ▶); data reduction: *DENZO* (Otwinowski & Minor, 1997 ▶) and *SCALEPACK*; program(s) used to solve structure: *SHELXS97* (Sheldrick, 2008 ▶); program(s) used to refine structure: *SHELXL97* (Sheldrick, 2008 ▶); molecular graphics: *PLATON* (Spek, 2009 ▶) and *ORTEPII* (Johnson, 1976 ▶); software used to prepare material for publication: *SHELXL97*.

## Supplementary Material

Crystal structure: contains datablocks global, I. DOI: 10.1107/S1600536811018022/gk2369sup1.cif

Structure factors: contains datablocks I. DOI: 10.1107/S1600536811018022/gk2369Isup2.hkl

Supplementary material file. DOI: 10.1107/S1600536811018022/gk2369Isup3.cml

Additional supplementary materials:  crystallographic information; 3D view; checkCIF report

## Figures and Tables

**Table 1:** Hydrogen-bond geometry (Å, °)

*D*—H⋯*A*	*D*—H	H⋯*A*	*D*⋯*A*	*D*—H⋯*A*
N8—H8⋯N14^i^	0.86	2.16	2.963 (2)	155
C5—H5⋯O21^ii^	0.93	2.54	3.308 (2)	140
C11—H11⋯O21^iii^	0.93	2.58	3.193 (2)	124
C16—H16⋯O21^iv^	0.98	2.52	3.492 (2)	171
C20—H20*A*⋯O21^ii^	0.96	2.58	3.412 (3)	145

Symmetry codes: (i) 
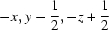
; (ii) 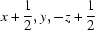; (iii) 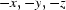; (iv) 
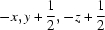
.

**Fig. 1.**
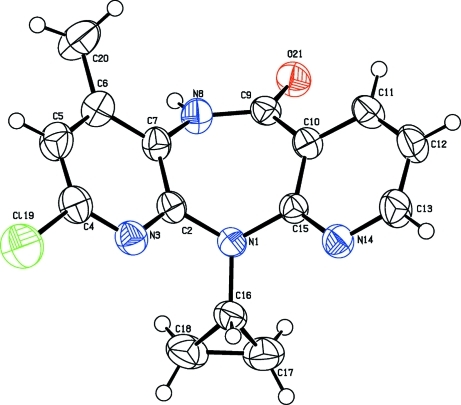
A view of the title compound with 50% probability displacement ellipsoids.

## supplementary crystallographic information

### Comment

The investigation of the chemistry of azepines continues to be an active area of heterocyclic chemistry (Le Count, 1996). The title diazepine compound is a chloro derivative  of the well known anti-HIV drug Nevirapine. The drug Nevirapine is the first human immunodeficiency virus type 1 (HIV-1) non-nucleoside reverse transcriptase (RT) inhibitor to reach regulatory approval. The title compound has been synthesized as a basic scaffold as reported earlier (Matsumoto *et al.*, 1984). We have also synthesized its derivatives and tested for secretory phospholipase A_2_ with anti-inflammatory activity (Thimmegowda *et al.*, 2007). We have identified a few derivatives with good activity. In view of this we have crystallized the title compound and finally the structure was confirmed by the X-ray diffraction studies.

A perspective view of the title molecule is shown in Fig. 1. The atoms N1 and N8 deviate -0.4953 (16) Å and -0.2666 (17) Å respectively with respect to the Cremer and Pople plane (Cremer & Pople, 1975) defined by the atoms C7/C9/C10/C15/C2 of the diazepine ring. The diazepine ring in the molecule adopts a twisted boat conformation as indicated by the puckering parameters *Q_2_* = 0.8015 (18) Å, *Q_3_* = 0.1262 (19) Å, φ_2_ = 182.80 (14)°, φ_3_ = 181.4 (9)°, and the total puckering amplitude Q_T_ = 0.8111 (18) Å. Chloromethylpyridine and pyridine units are planar with a maximum deviation of -0.015 (2) Å and 0.019 (2) Å for the atoms C7 and C12, respectively. As a result of the twisted boat conformation of the diazepine ring the molecule as a whole adopts a butterfly shape which is essential for the association of the inhibition pocket. The dihedral angle between the least squares planes of the pyridine N3/C4/C5/C6/C7/C2 and the best plane of the seven membered diaazepine ring C7/N8/C9/C10/C15/N1/C2 is 30.23 (9)°, and that between the diazepine ring and the pyridine ring C10/C11/C12/C13/N14/C15 is 28.49 (9)°.

The pyridine and the keto group at C10 and C9 are *gauche* oriented with respect to each other as indicated by the C11—C10—C9—O21 torsion angle value of 32.8 (3)° . The C15—N1—C16—C17  torsion angle for the cyclopropyl ring of -77.4 (2)°  indicates that the cyclopropyl ring is in equatorial position with respect to the diazepine ring. This value is low when compared to the corresponding value of 90.60 (2)° reported earlier (Thimmegowda *et al.*, 2008). The C9–N8 = 1.352 (2) Å bond length in the seven membered ring system is longer than a typical C=N bond (1.28 Å), but shorter than the C–N bond (C–N = 1.47 Å). The bond lengths N1–C15 = 1.416 (2) Å, N1–C2 =1.419 (2) Å, C7–N8 = 1.413 (2) Å indicates π-electron delocalization in the ring. As a result of the difference in the environment, there is a difference in the C–N bond lengths in the diazepine ring (C9–N8 = 1.352 (2)Å and C7–N8 = 1.413 (2) Å).

The structure exhibits intermolecular hydrogen bonds of the N—H···N and C—H···O type. The oxygen atom attatched to the diazepine ring accepts four intermolecular C—H···O hydrogen bonds.

### Experimental

The title compound was synthesized as per the procedure reported earlier (Matsumoto *et al.*, 1984). After synthesis and purification, the resultant pure product obtained was dissolved in ethyl acetate and was left undisturbed for slow evaporation of the solvent. Brown crystals grew after five days.

### Refinement

H atoms were placed at idealized positions and allowed to ride on their parent atoms with C–H distances in the range 0.93–0.98 Å and N-H distance 0.86 Å; *U*_iso_(H) = 1.2*U*_eq_(carrier atom) for all H atoms.

### Crystal data

**Table:**

C_15_H_13_ClN_4_O	*F*(000) = 1248
*M**_r_* = 300.74	*D*_x_ = 1.396 Mg m^−^^3^
Orthorhombic, *P**b**c**a*	Mo *K*α radiation, λ = 0.71073 Å
Hall symbol: -P 2ac 2ab	Cell parameters from 4721 reflections
*a* = 12.7750 (6) Å	θ = 2.5–25.0°
*b* = 13.5870 (7) Å	µ = 0.27 mm^−^^1^
*c* = 16.4920 (9) Å	*T* = 293 K
*V* = 2862.6 (3) Å^3^	Block, brown
*Z* = 8	0.27 × 0.25 × 0.25 mm

### Data collection

**Table:**

MacScience DIPLabo 32001 diffractometer	2155 reflections with *I* > 2σ(*I*)
Radiation source: fine-focus sealed tube	*R*_int_ = 0.014
graphite	θ_max_ = 25.0°, θ_min_ = 3.2°
Detector resolution: 10.0 pixels mm^-1^	*h* = −15→15
ω scans	*k* = −16→16
4721 measured reflections	*l* = −19→19
2525 independent reflections	

### Refinement

**Table:**

Refinement on *F*^2^	Secondary atom site location: difference Fourier map
Least-squares matrix: full	Hydrogen site location: inferred from neighbouring sites
*R*[*F*^2^ > 2σ(*F*^2^)] = 0.040	H-atom parameters constrained
*wR*(*F*^2^) = 0.114	*w* = 1/[σ^2^(*F*_o_^2^) + (0.059*P*)^2^ + 1.1189*P*] where *P* = (*F*_o_^2^ + 2*F*_c_^2^)/3
*S* = 1.03	(Δ/σ)_max_ = 0.013
2525 reflections	Δρ_max_ = 0.34 e Å^−^^3^
192 parameters	Δρ_min_ = −0.39 e Å^−^^3^
0 restraints	Extinction correction: *SHELXL97* (Sheldrick, 2008), FC^*^=KFC[1+0.001XFC^2^Λ^3^/SIN(2Θ)]^-1/4^
Primary atom site location: structure-invariant direct methods	Extinction coefficient: 0.0027 (7)

### Special details

**Table:**

Geometry. Bond distances, angles *etc*. have been calculated using the rounded fractional coordinates. All su's are estimated from the variances of the (full) variance-covariance matrix. The cell e.s.d.'s are taken into account in the estimation of distances, angles and torsion angles
Refinement. Refinement on *F*^2^ for ALL reflections except those flagged by the user for potential systematic errors. Weighted *R*-factors *wR* and all goodnesses of fit *S* are based on *F*^2^, conventional *R*-factors *R* are based on *F*, with *F* set to zero for negative *F*^2^. The observed criterion of *F*^2^ > σ(*F*^2^) is used only for calculating -*R*-factor-obs *etc*. and is not relevant to the choice of reflections for refinement. *R*-factors based on *F*^2^ are statistically about twice as large as those based on *F*, and *R*-factors based on ALL data will be even larger.

### Fractional atomic coordinates and isotropic or equivalent isotropic displacement parameters (Å^2^)

**Table:**

	*x*	*y*	*z*	*U*_iso_*/*U*_eq_	
Cl19	0.26118 (5)	0.13670 (5)	0.51234 (4)	0.0776 (3)	
O21	0.00487 (10)	−0.08660 (9)	0.11274 (8)	0.0483 (4)	
N1	−0.00616 (11)	0.11150 (10)	0.29568 (8)	0.0364 (4)	
N3	0.12574 (12)	0.11681 (11)	0.39490 (9)	0.0434 (5)	
N8	0.07762 (12)	−0.06760 (11)	0.23540 (9)	0.0421 (5)	
N14	−0.01401 (12)	0.23835 (11)	0.19903 (9)	0.0445 (5)	
C2	0.08540 (13)	0.07028 (12)	0.33068 (10)	0.0360 (5)	
C4	0.21102 (16)	0.07779 (15)	0.42695 (12)	0.0490 (6)	
C5	0.26156 (16)	−0.00449 (16)	0.39890 (12)	0.0524 (7)	
C6	0.22027 (15)	−0.05343 (14)	0.33216 (12)	0.0462 (6)	
C7	0.12823 (14)	−0.01540 (12)	0.29831 (10)	0.0378 (5)	
C9	0.03801 (13)	−0.03110 (13)	0.16565 (10)	0.0365 (5)	
C10	0.03435 (13)	0.07739 (13)	0.15357 (10)	0.0371 (5)	
C11	0.04651 (16)	0.11305 (14)	0.07545 (11)	0.0449 (6)	
C12	0.03218 (17)	0.21156 (15)	0.05952 (12)	0.0521 (7)	
C13	0.00047 (17)	0.27026 (15)	0.12274 (12)	0.0530 (7)	
C15	0.00547 (13)	0.14368 (12)	0.21449 (10)	0.0344 (5)	
C16	−0.07237 (15)	0.16871 (13)	0.34905 (10)	0.0419 (5)	
C17	−0.18729 (17)	0.15434 (18)	0.33813 (13)	0.0600 (7)	
C18	−0.13242 (19)	0.11117 (17)	0.41005 (13)	0.0626 (8)	
C20	0.27121 (18)	−0.14459 (17)	0.29923 (18)	0.0685 (8)	
H5	0.32220	−0.02710	0.42400	0.0630*	
H8	0.07130	−0.13010	0.24230	0.0510*	
H11	0.06440	0.07030	0.03370	0.0540*	
H12	0.04350	0.23730	0.00800	0.0630*	
H13	−0.01180	0.33650	0.11200	0.0640*	
H16	−0.04800	0.23440	0.36480	0.0500*	
H17A	−0.21030	0.10970	0.29580	0.0720*	
H17B	−0.23270	0.21050	0.34720	0.0720*	
H18A	−0.14470	0.14120	0.46250	0.0750*	
H18B	−0.12240	0.04040	0.41120	0.0750*	
H20A	0.33340	−0.15860	0.32980	0.1030*	
H20B	0.28930	−0.13450	0.24330	0.1030*	
H20C	0.22350	−0.19900	0.30350	0.1030*	

### Atomic displacement parameters (Å^2^)

**Table:**

	*U*^11^	*U*^22^	*U*^33^	*U*^12^	*U*^13^	*U*^23^
Cl19	0.0725 (4)	0.0946 (5)	0.0656 (4)	0.0017 (3)	−0.0270 (3)	−0.0291 (3)
O21	0.0605 (9)	0.0418 (7)	0.0425 (7)	0.0021 (6)	−0.0068 (6)	−0.0094 (6)
N1	0.0424 (8)	0.0363 (8)	0.0306 (7)	0.0038 (6)	0.0007 (6)	0.0004 (6)
N3	0.0483 (9)	0.0435 (9)	0.0384 (8)	−0.0026 (7)	−0.0034 (7)	−0.0029 (6)
N8	0.0541 (9)	0.0305 (7)	0.0417 (8)	−0.0012 (6)	−0.0085 (7)	−0.0020 (6)
N14	0.0589 (9)	0.0352 (8)	0.0393 (8)	0.0042 (7)	0.0000 (7)	0.0018 (6)
C2	0.0397 (9)	0.0355 (9)	0.0327 (8)	−0.0036 (7)	−0.0003 (7)	0.0023 (7)
C4	0.0498 (11)	0.0538 (12)	0.0435 (10)	−0.0054 (9)	−0.0078 (9)	−0.0036 (9)
C5	0.0459 (11)	0.0594 (12)	0.0520 (11)	0.0021 (9)	−0.0134 (9)	−0.0010 (10)
C6	0.0451 (10)	0.0427 (10)	0.0508 (11)	0.0018 (8)	−0.0051 (8)	0.0015 (8)
C7	0.0435 (9)	0.0337 (9)	0.0363 (9)	−0.0023 (7)	−0.0044 (7)	0.0027 (7)
C9	0.0378 (9)	0.0380 (9)	0.0338 (9)	0.0012 (7)	0.0005 (7)	−0.0042 (7)
C10	0.0389 (9)	0.0388 (9)	0.0337 (9)	−0.0011 (7)	−0.0017 (7)	−0.0010 (7)
C11	0.0526 (11)	0.0495 (11)	0.0326 (9)	−0.0007 (9)	0.0012 (8)	−0.0032 (8)
C12	0.0684 (13)	0.0528 (11)	0.0351 (10)	−0.0018 (10)	0.0012 (9)	0.0085 (9)
C13	0.0744 (14)	0.0393 (11)	0.0454 (11)	0.0031 (9)	0.0005 (10)	0.0091 (8)
C15	0.0373 (9)	0.0336 (8)	0.0324 (8)	−0.0004 (7)	−0.0018 (7)	0.0000 (7)
C16	0.0513 (10)	0.0386 (9)	0.0359 (9)	0.0038 (8)	0.0054 (8)	−0.0023 (7)
C17	0.0479 (11)	0.0705 (14)	0.0615 (13)	0.0062 (10)	0.0079 (10)	−0.0075 (11)
C18	0.0743 (15)	0.0587 (13)	0.0549 (13)	0.0029 (11)	0.0254 (11)	0.0067 (10)
C20	0.0592 (13)	0.0570 (13)	0.0892 (17)	0.0176 (11)	−0.0199 (12)	−0.0173 (13)

### Geometric parameters (Å, °)

**Table:**

Cl19—C4	1.742 (2)	C10—C11	1.385 (2)
O21—C9	1.229 (2)	C11—C12	1.376 (3)
N1—C2	1.420 (2)	C12—C13	1.374 (3)
N1—C15	1.416 (2)	C16—C18	1.487 (3)
N1—C16	1.447 (2)	C16—C17	1.492 (3)
N3—C2	1.337 (2)	C17—C18	1.497 (3)
N3—C4	1.322 (3)	C5—H5	0.9300
N8—C7	1.413 (2)	C11—H11	0.9300
N8—C9	1.351 (2)	C12—H12	0.9300
N14—C13	1.344 (2)	C13—H13	0.9300
N14—C15	1.335 (2)	C16—H16	0.9800
N8—H8	0.8600	C17—H17A	0.9700
C2—C7	1.393 (2)	C17—H17B	0.9700
C4—C5	1.371 (3)	C18—H18A	0.9700
C5—C6	1.390 (3)	C18—H18B	0.9700
C6—C20	1.501 (3)	C20—H20A	0.9600
C6—C7	1.400 (3)	C20—H20B	0.9600
C9—C10	1.488 (2)	C20—H20C	0.9600
C10—C15	1.399 (2)		
			
Cl19···H17B^i^	3.1100	C7···H13^iv^	2.9100
O21···C12^ii^	3.343 (2)	C7···H17A^vii^	3.0900
O21···C11^ii^	3.193 (2)	C13···H8^v^	2.7600
O21···C20^iii^	3.412 (3)	C15···H17A	3.1000
O21···C5^iii^	3.308 (2)	C20···H8	2.7300
O21···H16^iv^	2.5200	H5···H20A	2.3700
O21···H11^ii^	2.5800	H5···H11^viii^	2.3900
O21···H11	2.6100	H5···O21^vii^	2.5400
O21···H5^iii^	2.5400	H8···C20	2.7300
O21···H20A^iii^	2.5800	H8···H20C	2.3800
N1···N8	2.838 (2)	H8···N14^iv^	2.1600
N3···C18	3.308 (3)	H8···C13^iv^	2.7600
N8···C13^iv^	3.365 (3)	H8···H13^iv^	2.5600
N8···N1	2.838 (2)	H8···H16^iv^	2.5700
N8···N14^iv^	2.963 (2)	H11···O21	2.6100
N14···C17	3.386 (3)	H11···H5^ix^	2.3900
N14···N8^v^	2.963 (2)	H11···O21^ii^	2.5800
N3···H12^vi^	2.9200	H12···N3^x^	2.9200
N3···H16	2.7800	H13···C7^v^	2.9100
N8···H20C	2.8100	H13···H8^v^	2.5600
N8···H20B	2.8600	H16···N3	2.7800
N14···H16	2.7700	H16···N14	2.7700
N14···H8^v^	2.1600	H16···O21^v^	2.5200
N14···H20C^v^	2.8100	H16···H8^v^	2.5700
C5···O21^vii^	3.308 (2)	H17A···C15	3.1000
C7···C13^iv^	3.589 (3)	H17A···C7^iii^	3.0900
C11···O21^ii^	3.193 (2)	H17B···Cl19^xi^	3.1100
C12···O21^ii^	3.343 (2)	H18B···C2	3.0000
C13···N8^v^	3.365 (3)	H20A···H5	2.3700
C13···C7^v^	3.589 (3)	H20A···O21^vii^	2.5800
C17···N14	3.386 (3)	H20B···N8	2.8600
C18···N3	3.308 (3)	H20C···N8	2.8100
C20···O21^vii^	3.412 (3)	H20C···H8	2.3800
C2···H18B	3.0000	H20C···N14^iv^	2.8100
			
C2—N1—C15	114.82 (13)	N1—C16—C18	115.50 (16)
C2—N1—C16	116.50 (13)	N1—C16—C17	115.56 (15)
C15—N1—C16	118.07 (13)	C17—C16—C18	60.35 (14)
C2—N3—C4	116.41 (16)	C16—C17—C18	59.67 (14)
C7—N8—C9	127.75 (15)	C16—C18—C17	59.98 (14)
C13—N14—C15	117.66 (16)	C4—C5—H5	121.00
C9—N8—H8	116.00	C6—C5—H5	121.00
C7—N8—H8	116.00	C10—C11—H11	120.00
N3—C2—C7	123.20 (16)	C12—C11—H11	120.00
N1—C2—N3	116.95 (14)	C11—C12—H12	121.00
N1—C2—C7	119.85 (15)	C13—C12—H12	121.00
Cl19—C4—C5	118.30 (16)	N14—C13—H13	118.00
Cl19—C4—N3	116.24 (15)	C12—C13—H13	118.00
N3—C4—C5	125.46 (18)	N1—C16—H16	118.00
C4—C5—C6	118.59 (19)	C17—C16—H16	118.00
C5—C6—C7	117.25 (17)	C18—C16—H16	118.00
C7—C6—C20	121.62 (18)	C16—C17—H17A	118.00
C5—C6—C20	121.12 (19)	C16—C17—H17B	118.00
N8—C7—C6	119.44 (15)	C18—C17—H17A	118.00
N8—C7—C2	121.41 (15)	C18—C17—H17B	118.00
C2—C7—C6	119.04 (16)	H17A—C17—H17B	115.00
O21—C9—C10	120.14 (15)	C16—C18—H18A	118.00
O21—C9—N8	120.57 (16)	C16—C18—H18B	118.00
N8—C9—C10	119.30 (15)	C17—C18—H18A	118.00
C11—C10—C15	118.19 (16)	C17—C18—H18B	118.00
C9—C10—C15	123.36 (15)	H18A—C18—H18B	115.00
C9—C10—C11	117.87 (15)	C6—C20—H20A	109.00
C10—C11—C12	120.19 (17)	C6—C20—H20B	110.00
C11—C12—C13	117.32 (18)	C6—C20—H20C	109.00
N14—C13—C12	124.33 (19)	H20A—C20—H20B	109.00
N1—C15—N14	117.29 (14)	H20A—C20—H20C	110.00
N14—C15—C10	122.17 (15)	H20B—C20—H20C	109.00
N1—C15—C10	120.52 (15)		
			
C15—N1—C2—N3	117.40 (16)	N3—C2—C7—N8	173.78 (16)
C15—N1—C2—C7	−63.2 (2)	N3—C2—C7—C6	−2.5 (3)
C16—N1—C2—N3	−26.7 (2)	Cl19—C4—C5—C6	178.23 (15)
C16—N1—C2—C7	152.68 (16)	N3—C4—C5—C6	−1.3 (3)
C2—N1—C15—N14	−121.30 (16)	C4—C5—C6—C7	−0.6 (3)
C2—N1—C15—C10	59.9 (2)	C4—C5—C6—C20	−179.2 (2)
C16—N1—C15—N14	22.2 (2)	C5—C6—C7—N8	−174.00 (17)
C16—N1—C15—C10	−156.65 (16)	C5—C6—C7—C2	2.4 (3)
C2—N1—C16—C17	−139.70 (16)	C20—C6—C7—N8	4.6 (3)
C2—N1—C16—C18	−72.0 (2)	C20—C6—C7—C2	−179.04 (18)
C15—N1—C16—C17	77.4 (2)	O21—C9—C10—C11	−32.9 (2)
C15—N1—C16—C18	145.11 (17)	O21—C9—C10—C15	138.19 (18)
C4—N3—C2—N1	−179.96 (16)	N8—C9—C10—C11	147.41 (17)
C4—N3—C2—C7	0.7 (3)	N8—C9—C10—C15	−41.5 (2)
C2—N3—C4—Cl19	−178.26 (13)	C9—C10—C11—C12	172.74 (18)
C2—N3—C4—C5	1.2 (3)	C15—C10—C11—C12	1.2 (3)
C9—N8—C7—C2	48.6 (3)	C9—C10—C15—N1	10.1 (2)
C9—N8—C7—C6	−135.18 (19)	C9—C10—C15—N14	−168.66 (16)
C7—N8—C9—O21	173.00 (16)	C11—C10—C15—N1	−178.83 (16)
C7—N8—C9—C10	−7.3 (3)	C11—C10—C15—N14	2.4 (3)
C15—N14—C13—C12	1.2 (3)	C10—C11—C12—C13	−3.3 (3)
C13—N14—C15—N1	177.66 (16)	C11—C12—C13—N14	2.2 (3)
C13—N14—C15—C10	−3.5 (3)	N1—C16—C17—C18	106.08 (18)
N1—C2—C7—N8	−5.5 (2)	N1—C16—C18—C17	−106.17 (18)
N1—C2—C7—C6	178.18 (16)		

Symmetry codes: (i) *x*+1/2, −*y*+1/2, −*z*+1; (ii) −*x*, −*y*, −*z*; (iii) *x*−1/2, *y*, −*z*+1/2; (iv) −*x*, *y*−1/2, −*z*+1/2; (v) −*x*, *y*+1/2, −*z*+1/2; (vi) *x*, −*y*+1/2, *z*+1/2; (vii) *x*+1/2, *y*, −*z*+1/2; (viii) −*x*+1/2, −*y*, *z*+1/2; (ix) −*x*+1/2, −*y*, *z*−1/2; (x) *x*, −*y*+1/2, *z*−1/2; (xi) *x*−1/2, −*y*+1/2, −*z*+1.

### Hydrogen-bond geometry (Å, °)

**Table:**

*D*—H···*A*	*D*—H	H···*A*	*D*···*A*	*D*—H···*A*
N8—H8···N14^iv^	0.86	2.16	2.963 (2)	155
C5—H5···O21^vii^	0.93	2.54	3.308 (2)	140
C11—H11···O21^ii^	0.93	2.58	3.193 (2)	124
C16—H16···O21^v^	0.98	2.52	3.492 (2)	171
C20—H20A···O21^vii^	0.96	2.58	3.412 (3)	145

Symmetry codes: (iv) −*x*, *y*−1/2, −*z*+1/2; (vii) *x*+1/2, *y*, −*z*+1/2; (ii) −*x*, −*y*, −*z*; (v) −*x*, *y*+1/2, −*z*+1/2.
